# Molecular Calcification Imaging and Ascending Aortic Disease in Patients With a Bicuspid Aortic Valve

**DOI:** 10.1001/jamanetworkopen.2025.60385

**Published:** 2026-02-23

**Authors:** Jennifer Nash, Samuel Debono, Krithika Loganath, Beth Whittington, Evangelos Tzolos, Maaz Syed, Laura Clark, Tijana Mitić, Adriana A. S. Tavares, Mark Macaskill, Stephanie L. Sellers, Tim Clark, Scott Semple, Gillian MacNaught, Edwin J. R. van Beek, Damini Dey, Piotr Slomka, Niki Walker, Jillian Madine, Mark Field, Riaz Akhtar, Rachael O. Forsythe, Michelle C. Williams, Marc R. Dweck, David E. Newby, Alexander J. Fletcher

**Affiliations:** 1 The University of Edinburgh Centre for Cardiovascular Science, University of Edinburgh, Edinburgh, United Kingdom; 2 Department of Radiology, Division of Cardiology, Cardiovascular Translational Lab at the Centre for Heart Lung Innovation, St Paul’s Hospital and University of British Columbia, Vancouver, Canada; 3 Edinburgh Imaging Facility Queens Medical Research Institute, University of Edinburgh, Edinburgh, United Kingdom; 4 Department of Medical Physics, NHS Lothian, Royal Infirmary of Edinburgh, Edinburgh, United Kingdom; 5 Division of Artificial Intelligence, Department of Medicine, and Biomedical Imaging Research Institute, Cedars-Sinai Medical Centre, Los Angeles, California; 6 Scottish Adult Congenital Cardiology Service, Golden Jubilee National Hospital, Clydebank, Glasgow, United Kingdom; 7 Institute of Systems, Molecular and Integrative Biology, Faculty of Health and Life Sciences, University of Liverpool, Liverpool, United Kingdom; 8 Department of Cardiothoracic Surgery, Liverpool Heart and Chest Hospital, Liverpool, United Kingdom; 9 Department of Mechanical, Materials and Aerospace Engineering, School of Engineering, University of Liverpool, Liverpool, United Kingdom; 10 School of Cardiovascular and Metabolic Health, University of Glasgow, Glasgow, United Kingdom

## Abstract

**Importance:**

Selection of patients with a bicuspid aortic valve and aortopathy for prophylactic aortic surgery remains challenging. In thoracic aortopathy, aortic medial elastin fiber fragmentation initially leads to microcalcification but later declines with progressive loss of elastin content and reduced structural integrity.

**Objective:**

To determine whether aortic microcalcification detected using fluorine F 18–labeled [18F]–sodium fluoride positron emission tomography (PET) is associated with future aortic diameter expansion.

**Design, Setting, and Participants:**

This prospective longitudinal cohort study was conducted in tertiary care centers across Scotland from April 4, 2019, to September 15, 2023. Participants included patients with a bicuspid aortic valve. Data analysis was performed from May 21, 2024, to March 4, 2025.

**Exposures:**

Hybrid [18F]–sodium fluoride PET and computed tomography.

**Main Outcomes and Measures:**

Baseline ascending aortic [18F]–sodium fluoride uptake was measured as mean tissue to background ratio. The primary outcome was ascending aortic diameter expansion during 24 months on cardiac magnetic resonance imaging (MRI).

**Results:**

Seventy-six patients with a bicuspid aortic valve (mean [SD] age, 52.6 [7.5] years; 57 [75.0%] male) underwent baseline [18F]–sodium fluoride PET and MRI. Fifty-six patients underwent follow-up MRI after a median of 723 (IQR, 515-787) days. There was an inverse correlation between baseline ascending aortic [18F]–sodium fluoride uptake and annual change in diameter (Pearson *r* = −0.37; *P* = .005), which remained after adjustment for confounders in multivariable regression analysis. Ascending aortic [18F]–sodium fluoride was not correlated with baseline diameter (Pearson *r* = 0.08; *P* = .50) but was moderately correlated with baseline ascending aortic stiffness index (Pearson *r* = 0.38; *P* < .001).

**Conclusion and Relevance:**

In this cohort study of patients with a bicuspid aortic valve, the most rapid aortic growth was seen in those with low [18F]–sodium fluoride ascending aortic uptake, indicating reduced aortic wall integrity. High ascending aortic [18F]–sodium fluoride uptake was associated with a stiffer and slow-growing ascending aortic phenotype. These findings suggest that [18F]–sodium fluoride PET imaging represents a promising new noninvasive approach to identify a microcalcified disease phenotype in thoracic aortopathy among patients with a bicuspid aortic valve.

## Key Points

**Question:**

Can fluorine F 18–labeled ([18F])–sodium fluoride positron emission tomography help identify a diseased aortic wall in patients with a bicuspid aortic valve?

**Findings:**

In this cohort study of 76 participants with a bicuspid aortic valve, low aortic wall [18F]–sodium fluoride uptake was associated with faster ascending aortic growth rate independent of baseline diameter. Higher [18F]–sodium fluoride uptake was also associated with a stiff and slow growing aortic wall phenotype.

**Meaning:**

These findings suggest that [18F]–sodium fluoride can identify disease activity within the ascending aorta, with the potential to improve patient selection for prophylactic aortic surgery.

## Teaser

This cohort study examines whether positron emission tomography imaging with [18F]–sodium fluoride can detect aortopathy, identify disease severity, and estimate disease progression in patients with a bicuspid aortic valve.

## Introduction

Thoracic aortopathy is a pathological process most frequently associated with a bicuspid aortic valve and is present in 0.5% of the population.^1,2,3^ While a clinically silent disease process, approximately 1 in 160 individuals with a bicuspid aortic valve will experience a life-threatening complication of acute Stanford type A aortic dissection, which has a 26% to 58% mortality rate.^4,5^ The primary management of bicuspid thoracic aortopathy is surgical replacement of the diseased aortic wall once the risk of dissection is deemed too great. The best-known risk factor for acute aortic dissection is extent of dilation or rapidly enlarging aortic diameters,^6^ with at-risk patients monitored through echocardiography, computed tomography (CT), or magnetic resonance imaging (MRI) surveillance programs. Once internationally agreed threshold diameters are met, patients are offered prophylactic aortic surgery.^7,8^ Despite these robust aortic surveillance programs, as many as three-quarters of patients with a bicuspid aortic valve who experience type A aortic dissection have aortic diameters below these surgical thresholds.^9^ There is therefore an urgent unmet clinical need to develop improved approaches to risk stratification in this large and vulnerable patient group.

Thoracic aortic microcalcification refers to lesions of hydroxyapatite (Ca_10_ [PO_4_]_6_ [OH]_2_) less than 50 µm in length deposited in the aortic wall and have been closely linked with thoracic aortic disease.^10,11,12^ In the early stages of thoracic aortopathy, microscopic calcification deposits on damaged elastin fibers within the media layer.^12,13^ This process is augmented by extracellular matrix damage, which promotes vascular smooth muscle cells switching to an osteoblastic phenotype with calcific vesicle secretion and further deposition of microcalcification onto fragmented elastin fibers.^14,15^ However, as the aortic disease progresses toward severe histological disease, there is a sharp decline of both elastin fibers and consequently of microcalcification, which is strongly adherent to the elastin, making microcalcification an attractive target that could differentiate severe disease from less vulnerable forms.^13^ The ability to quantify the microcalcification content within the aortic wall could allow pathological disease progression to be tracked noninvasively over time. In the present study, we aim to translate these findings in a prospective molecular imaging study examining whether positron emission tomography (PET) imaging with fluorine F 18–labeled ([18F])–sodium fluoride, a radiotracer that binds with hydroxyapatite crystals, could detect aortopathy, identify disease severity, and those with a bicuspid aortic valve most likely to experience disease progression.

## Methods

### Study Population

For this cohort study, patients with a bicuspid aortic valve were identified from cardiology clinics across Scotland between April 4, 2019, and July 24, 2021, with follow-up completed on September 15, 2023. Inclusion criteria were age older than 40 years with a bicuspid aortic valve confirmed on echocardiography or MRI. Exclusion criteria were previous or planned aortic replacement surgery or aortic valve surgery, inability to tolerate MRI, contrast allergy, estimated glomerular filtration rate less than 30 mL/min/1.73 m^2^, pregnancy, or known connective tissue disease (eFigure in Supplement 1). The study was approved by the local institutional review board, the Scottish Research Ethics Committee, and the United Kingdom Administration of Radiation Substances Advisory Committee. Written informed consent was obtained from all participants. This study followed the Strengthening the Reporting of Observational Studies in Epidemiology (STROBE) reporting guideline and was registered on ClinicalTrials.gov (NCT04083118).

### Study Design

This was a single-center prospective cohort study. At the baseline visit, all participants underwent a structured clinical interview, clinical examination including 3 measurements of brachial blood pressure, applanation tonometry, and [18F]–sodium fluoride PET-CT, followed immediately by MRI. Demographic data were confirmed in the structured interview. Hypertension, type 2 diabetes, and coarctation status were ascertained as part of the prospective structured interview and confirmed with retrospective review of the clinical notes. Regular exercise was defined as exercise or sporting activity of at least moderate intensity on European guidelines at least weekly on average.^16^ Smoking status was reported by the patient at the time of the structured interview. Participants who had smoked cigarettes at least daily for longer than 1 month at some point in their lifetime were identified as smokers. Former smokers had smoked cigarettes previously but not in the last month. Medication use was confirmed by the patient at the time of structured interview. Applanation tonometry was performed using a commercially available system (Sphygmacor; AtCor Medical Pty Ltd), which mathematically derived estimates of central aortic pressure from radially acquired pulse waveforms that correlate well with invasive assessment. Patients undertook a second visit at 24 months after their initial visit, which included a structured clinical interview, clinical examination, applanation tonometry, and MRI.

### Image Acquisition

Participants received an injection of 250 MBq of [18F]–sodium fluoride and after 60 minutes underwent PET-CT in a hybrid 128-detector array scanner (Biograph mCT, Siemens Healthineers). Low-dose attenuation-correction CT was performed (120 kV, 5/3 mm) with PET acquisition in three 10-minute bed positions to cover the whole aorta. The PET images were reconstructed using the ultra–high-definition algorithm (256 matrix, 4 iterations, 5-mm gaussian filter, 21 subsets).

Patients were then immediately transferred for MRI scanning on a 3T device (Biograph mMR). Images were acquired using electrocardiogram gating. The imaging protocol included 2-, 3-, and 4-chamber long-axis, short-axis stack, axial aortic (at the level of the right pulmonary artery) steady-state free procession cine images. The aortic valve was assessed using left-ventricular outflow tract, aortic valve cine images, and 2-dimensional phase contrast images at the sinotubular junction with the velocity-encoded threshold set to the lowest value without aliasing. A gadolinium-enhanced contrast angiogram was performed at 1.5-mm section thickness.

### Image Analysis

We assessed [18F]–sodium fluoride uptake in the ascending aorta using dedicated PET image analysis FusionQuant software, version 1.21.0421 (Cedars-Sinai Medical Centre), and a previously published method with high interrater (interclass correlation coefficient, 0.97) and scan-rescan (interclass correlation coefficient, 0.86) repeatability.^17^ PET images were coregistered with the MR angiogram in 3 orthogonal planes via multiplanar reformat. The mean tissue to background ratio (TBR) was measured by drawing a volume of interest around the ascending aorta from the sinotubular junction to the innominate artery. The background activity was determined as the mean standardized uptake value of two 8-mm spheres, one in each of the right and left atrium.

MRI analysis was performed on dedicated CVI 42 software, version 5.11.2 (Circle Cardiovascular Imaging Inc). The ascending aortic diameter was measured at end-diastole at the level of the right pulmonary artery on aortic axial cine images^18^ by a trained user (J.N.) blinded to the PET quantification data.^18^ Aortic diameters were indexed to body surface area calculated using DuBois equation.^19^ Aortic dilation was defined as aortic diameter greater than the 95th percentile for age and sex and indexed for body surface area for the ascending aorta based on MRI reference values.^18^ Aortic valve anatomy was assessed using aortic valve cine images and classified using international consensus classification.^20^ Valve stenosis was measured using peak aortic jet velocity on 2-dimensional phase contrast images and graded as none (<2.0 m/s), mild (2.0-2.9 m/s), moderate (3.0-3.9 m/s), or severe (≥4.0 m/s).^21^ Valve regurgitation was graded as none, mild, moderate, or severe based on visual assessment of the valve, the presence or absence of flow reversal in the thoracic aorta, and regurgitation fraction by an accredited specialist (M.R.D.) with expertise in MRI blinded to the PET results (calculated using 2-dimensional phase contrast sequences and stroke volume differential). The circumferential ascending aortic stiffness index was used as it best accounts for distortions associated with aortic diameter.^22^ Ascending aortic stiffness index was calculated by taking the natural logarithm of the systolic-to-diastolic aortic blood pressure ratio and dividing by the circumferential ascending aortic strain.

The aortic systolic and diastolic pressure were taken from applanation tonometry results performed during the same study visit. Ascending aortic growth rate analysis was performed by a trained operator (J.N.) blind to the PET quantification. The growth rate per year was performed by subtracting the baseline measurement from the follow-up measurement and dividing by years of follow-up (number of days/365).

### Statistical Analysis

Data were analyzed from May 21, 2024, to March 4, 2025. Statistical analysis was performed using the software package R, version 4.0.2 (R Project for Statistical Computing). Shapiro-Wilk tests and histogram plots were used to assess normality of distribution of results. Categorical variables are presented as number (percentage); continuous variables with normal distribution are presented as mean (SD), and nonnormally distributed variables are presented as median (IQR). Associations between ascending aortic [18F]–sodium fluoride PET-CT uptake and aortic diameter at baseline were determined using the Pearson correlation coefficient and linear regression. Candidate variables for all regression analysis included cardiovascular risk factors (age, sex, smoking status, hypertension, and diabetes) and factors influencing ascending aortic hemodynamics (ascending aortic size indexed to body surface area, presence of an aneurysm [diameter >95th percentile] after adjustment for age, sex, and body surface, aortic valve stenosis or regurgitation severity, subtype of bicuspid aortic valve, coarctation status, and the ascending aortic calcium score). As no baseline variables were associated with ascending aortic [18F]–sodium fluoride uptake at baseline, a multivariable model for this variable was not constructed. For the primary outcome of annualized change in aortic size (in millimeters per year) as the dependent variable, a univariable linear regression analysis was performed using the same variables. Both coarctation status and baseline [18F]-sodium fluoride PET-CT uptake were statistically significantly associated with annualized ascending growth (*P* < .05) and were therefore used in a multivariable model. A final linear regression analysis was performed with baseline ascending aortic stiffness index as the dependent variable and the same dependent variables. Variables significant on univariable analysis (age, baseline aortic diameter, diabetes, and baseline ascending aortic [18F]–sodium fluoride mean TBR) were included in the multivariable model. Statistical significance was set as a 2-sided *P* < .05.

## Results

### Overall Cohort

**Figure 1.**
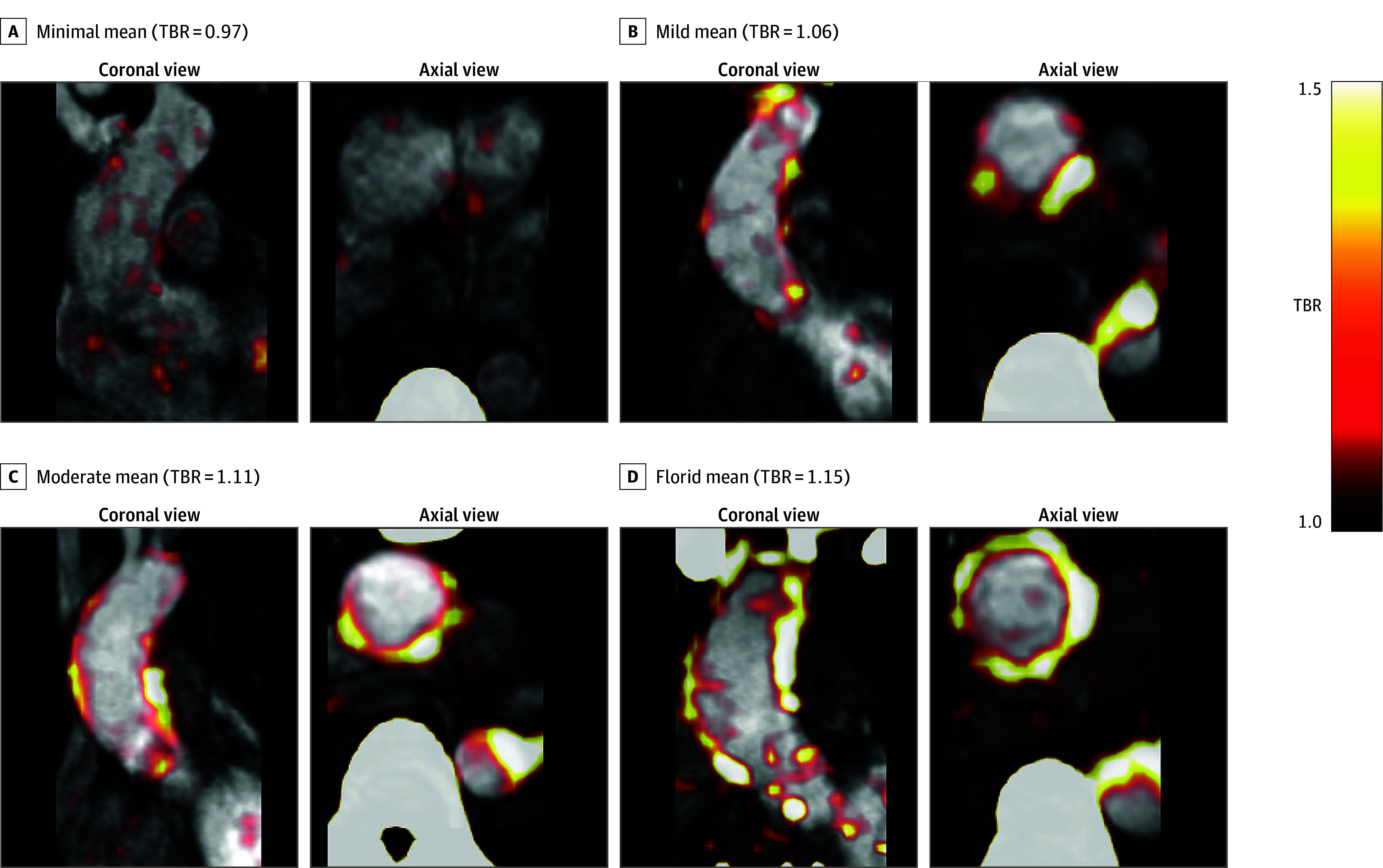
Fluorine F 18–Labeled ([18F])–Sodium Fluoride Uptake in the Ascending Aorta Coronal and axial [18F]–sodium fluoride positron emission tomography with computed tomography attenuation correction fused with magnetic resonance angiogram. Examples are shown for minimal (A), mild (B), moderate (C), and florid (D) ascending aortic [18F]–sodium fluoride uptake. Each positron emission image is scaled to a tissue background ratio of 1 to 1.5. TBR indicates tissue to background ratio.

Seventy-six patients with a bicuspid aortic valve (mean [SD] age, 52.6 [7.5] years; 57 [75.0%] male and 19 [25.0%] female) were recruited (Table and eFigure in Supplement 1). Visually, ascending aortic [18F]–sodium fluoride uptake was seen in most patients with varying intensity (Figure 1). There were no demonstratable associations between baseline [18F]–sodium fluoride uptake and demographic characteristics, cardiovascular risk factors, aortic valve stenosis, or regurgitation or bicuspid aortic valve subtype (eTable 1 in Supplement 1). Furthermore, baseline [18F]–sodium fluoride uptake was independent of ascending aortic size index or the presence of an associated ascending aortic aneurysm (eTable 2 in Supplement 1).

**Table.** Baseline Patient Characteristics

Characteristic	No. (%) of participants (N = 76)
Age, mean (SD), y	52.6 (7.5)
Sex	
Female	19 (25.0)
Male	57 (75.0)
Height, mean (SD), m	1.75 (0.10)
Weight, mean (SD), kg	86.3 (14.9)
Body surface area, mean (SD), m^2^	2.01 (0.20)
Cardiovascular risk factors	
Hypertension	32 (42.1)
Diabetes	3 (3.9)
Coarctation	19 (25.0)
Smoking status	
Current	9 (11.8)
Former	18 (23.7)
Never	49 (64.5)
Regular exercise	35 (46.1)
Family history of dissection	3 (3.9)
Antihypertensive medication	
Angiotensin receptor blocker	11 (14.5)
Angiotensin-converting enzyme inhibitor	21 (27.6)
β-Blocker	14 (18.4)
Aortic diameter	
Ascending aorta diameter, median (IQR), mm	38.4 (35.0-41.9)
Body surface area–indexed ascending aorta diameter, median (IQR), mm/m^2^	18.8 (16.9-21.5)
Age- and sex-adjusted body surface area–indexed ascending aortic diameter >95th percentile	23 (30.3)
Aortic stenosis grade	
None	44 (57.9)
Mild	21 (27.6)
Moderate	11 (14.5)
Aortic regurgitation grade	
None	44 (57.9)
Mild	15 (19.7)
Moderate	11 (14.5)
Severe	6 (7.9)
Bicuspid aortic valve subtype	
Right-left fusion	52 (68.4)
Right noncoronary cusp fusion	12 (15.8)
Left noncoronary cusp fusion	1 (1.3)
Two sinus type A	9 (11.8)
Two sinus type B	2 (2.6)

### Longitudinal Cohort

**Figure 2.**
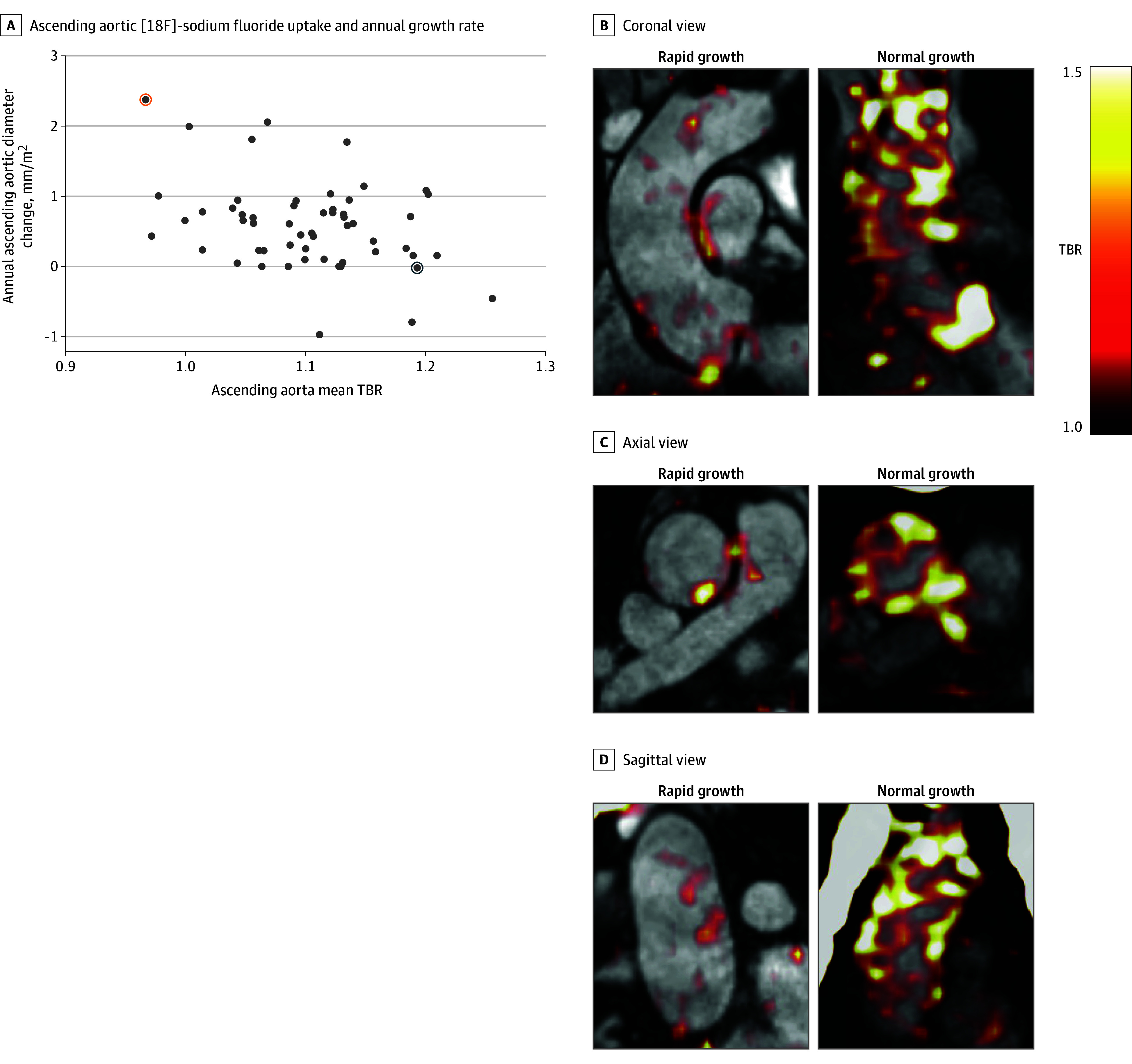
Baseline Ascending Aortic Fluorine F 18–Labeled ([18F])–Sodium Fluoride and Future Growth A, There was a moderate inverse correlation between baseline ascending aortic [18F]–sodium fluoride uptake and annual ascending aortic diameter growth rate (Pearson *r* = −0.37; *P* = .005). B-D, [18F]–Sodium fluoride positron emission tomography fused with magnetic resonance angiography in 2 participants. The left-hand images are from a patient in their 60s with a baseline ascending aortic diameter of 37 mm, moderate aortic regurgitation, and no aortic stenosis and taking an angiotensin-converting enzyme inhibitor for hypertension who demonstrated rapid ascending aortic growth (2 mm/y) and low baseline ascending aortic [18F]–sodium fluoride uptake (orange circle in panel A) (mean tissue to background ratio [TBR] = 0.97). The right-hand images are from a patient in their 40s with a baseline ascending aortic diameter of 41 mm, no ascending aortic growth, moderate aortic regurgitation and stenosis, and taking no regular medication who demonstrated no ascending aortic growth and high baseline ascending aortic [18F]–sodium fluoride uptake (blue circle in panel A) (mean TBR = 1.19). Each positron emission image is scaled to a tissue background ratio of 1 to 1.5.

Fifty-six patients with a bicuspid aortic valve underwent repeat MRI at a median of 723 (IQR, 515-787) days and were included in analysis of the primary outcome. There was no difference in baseline characteristics between those who received follow-up MRI and those who did not (eTable 2 in Supplement 1). The median annual change in diameter was 0.6 (IQR, 0.2-0.8) mm with a range of −1.0 to 2.4 mm. There was an inverse correlation between baseline ascending aortic [18F]–sodium fluoride uptake and annual change in diameter (*r* = −0.37; *P* = .005) (Figure 2). This association persisted after linear regression adjustment for the presence of coarctation (β = 0.70; 95% CI, 0.55-0.88; *P* = .004) (eTable 3 in Supplement 1). Age, sex, hypertension, diabetes, smoking status, aortic valve morphology, aortic stenosis, aortic regurgitation, baseline ascending aortic diameter, and baseline ascending aortic calcium score were not associated with diameter progression (eTable 3 in Supplement 1).

### Associations With Aortic Remodeling

**Figure 3.**
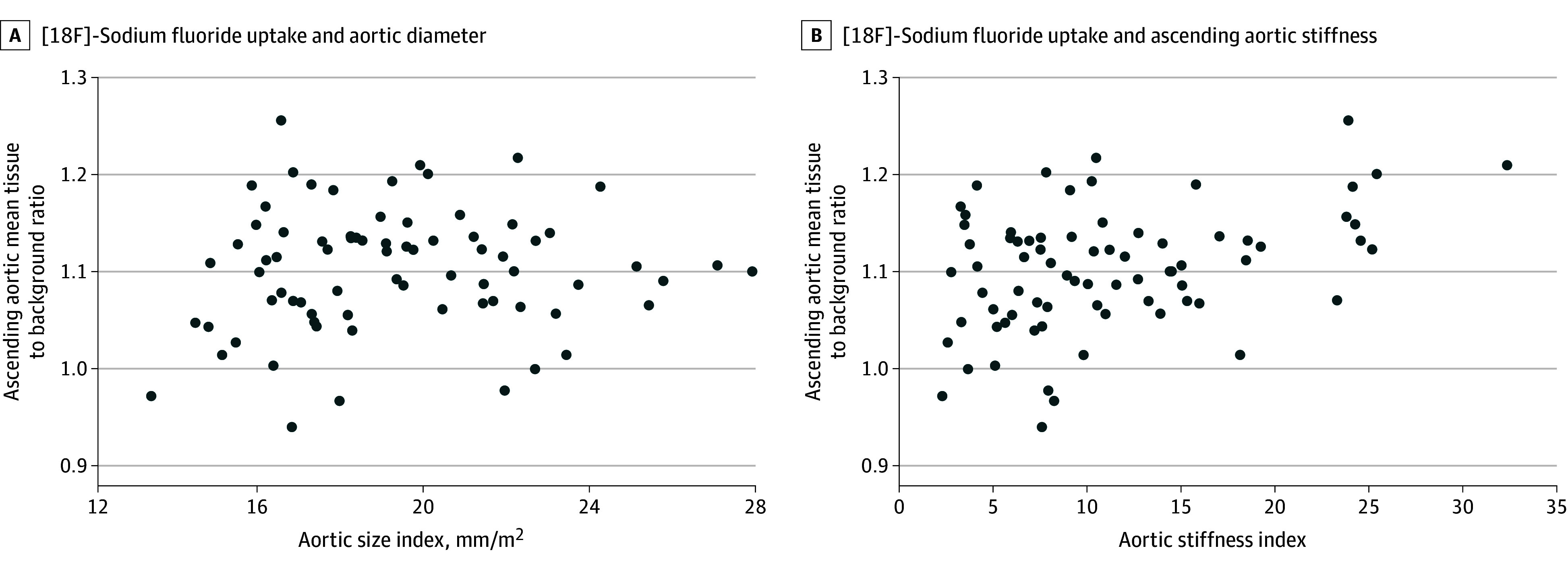
Ascending Aortic Fluorine F 18–Labeled ([18F])–Sodium Fluoride and Markers of Aortic Remodeling in Patients With a Bicuspid Aortic Valve A, There was no correlation between [18F]–sodium fluoride uptake and aortic diameter (Pearson *r* = 0.08; *P* = .50). B, There was an association between increasing baseline ascending aortic stiffness index (calculated by taking the natural logarithm of the systolic-to-diastolic aortic blood pressure ratio and dividing by the circumferential ascending aortic strain) and [18F]–sodium fluoride uptake (Pearson *r* = 0.38; *P* < .001).

There were no differences in ascending aortic [18F]–sodium fluoride uptake in patients with (median TBR-mean, 1.09; [IQR, 1.06-1.12]) or without (median TBR-mean, 1.12 [IQR, 1.05-1.14]; *P* = .34) ascending aortic dilatation (eTable 4 in Supplement 1). There was no correlation between ascending aortic diameter adjusted for body surface area and [18F]–sodium fluoride uptake (Pearson *r* = 0.08; *P* = .50) (Figure 3A).

There was a moderate correlation between ascending aortic [18F]–sodium fluoride uptake and baseline ascending aortic stiffness index (Pearson *r* = 0.38 [*P* < .001]; β = 1.51 [95% CI, 1.19-1.90; *P* < .001]) (Figure 3B and eTable 5 in Supplement 1). This association remained after adjustment for age, diabetes status, and ascending aortic diameter (β = 1.33; 95% CI, 1.08-1.62; *P* = .007) (eTable 5 in Supplement 1), all of which were associated with ascending aortic stiffness index on univariable linear regression (eTable 5 in Supplement 1).

## Discussion

To our knowledge, this is the first molecular imaging study to explore the potential of [18F]–sodium fluoride PET-CT in noninvasive identification and assessment of thoracic aortopathy. High baseline ascending aortic [18F]–sodium fluoride uptake was associated with biomechanical markers of aortic wall remodeling and stable aortic diameters. However, low ascending aortic [18F]–sodium fluoride uptake was associated with greater aortic diameter expansion rates, indicative of progressive loss of elastin fibers and worse structural integrity. These findings have the potential to provide a risk marker of future disease progression and dissection risk.

We did not observe an association between baseline [18F]–sodium fluoride uptake and baseline ascending aortic diameter. This result supports the notion that [18F]–sodium fluoride PET identifies a diameter-independent pathological process in patients with undilated or modestly dilated ascending aortic diameters. It should be highlighted that our selection criteria excluded those who were at threshold for aortic surgery and are most likely to have loss of elastin fibers and the associated loss of microcalcification due to advanced disease. Our findings are therefore confined to patients with undilated or modestly dilated ascending aortas, who are arguably the most crucial population in whom to identify those with active aortic disease and at risk.

Our primary outcome measure examined the association between baseline ascending aortic [18F]–sodium fluoride uptake and subsequent diameter progression. We found that baseline ascending aortic [18F]–sodium fluoride uptake was moderately and inversely correlated with the rate of ascending aortic expansion. Histological data demonstrate a “rise then fall” pattern of aortic microcalcification with increasing aortopathy severity.^13^ In keeping with this pattern, patients with high ascending aortic [18F]–sodium fluoride uptake had stable aortic diameters, while those with low uptake exhibited rapid aortic diameter expansion, suggesting a declining stage of aortopathy that may warrant closer follow-up. Long-term follow-up of the ascending aortic outcomes of this cohort, as well as repeated [18F]–sodium fluoride PET assessments, are now required to determine the potential clinical utility of [18F]–sodium fluoride.

To better understand the biomechanics of those with a microcalcified ascending aortic phenotype, we assessed the association between baseline ascending aortic [18F]–sodium fluoride uptake and robustly assessed localized stiffness index. We found a moderate correlation between baseline ascending aortic [18F]–sodium fluoride uptake and ascending aortic stiffness index that was present after adjusting for age, baseline diameter, and diabetes status, all of which are known to affect aortic stiffness. These data corroborate ex vivo results in which high aortic wall medial microcalcification was associated with increased stiffness as determined by results of nanoindentation testing.^13^ Results of studies linking aortic stiffness index and aortic growth rates in patients with a variety of aortopathies are mixed.^3,23,24,25,26,27,28^ Importantly, our findings are consistent with a metholodogically similar study that links high localized midascending aortic stiffness with slower growth rates.^28^

### Limitations

Our study has some limitations that should be acknowledged. This was a single-center cohort study, thus our findings are limited by the relatively small numbers of study participants. External validation in larger cohorts is now needed. Disease progression was based on rates of aortic expansion rather than clinical events. Our population was too small to assess associations with clinical outcomes, but the rate of aortic diameter expansion is a reasonable and intuitive marker of disease activity. Our follow-up duration was limited to 2 years, and further longitudinal surveillance may capture those who go on to experience aortic dissection or aortic replacement surgery. In addition, [18F]–sodium fluoride uptake cannot distinguish atherosclerotic calcification from medial calcification. However, the ascending aorta is known to be relatively spared from atherosclerosis,^29,30,31,32^ and indeed in histological study of patients undergoing ascending aortic replacement, all [18F]–sodium fluoride uptake arose from the media rather than representing intimal atherosclerosis.^13^ There remain questions as to the feasibility of routine clinical PET imaging in this cohort, given the associated cost and limited availability of PET imaging as well as the cumulative radiation exposure, especially for a relatively young patient group. However, [18F]–sodium fluoride is relatively inexpensive and widely available in clinical practice. A detection and surveillance imaging strategy is therefore plausible and deliverable within acceptable health care costs and patient safety concerns.

## Conclusions

The findings of this cohort study of patients with a bicuspid aortic valve suggest that [18F]–sodium fluoride imaging could identify patients with a microcalcified aortic wall phenotype that is independent of ascending aortic diameter. Importantly, patients with low ascending aortic [18F]–sodium fluoride uptake were at the highest risk for rapid progression of aortic diameters, indicating reduced aortic wall structural integrity. Future follow-up of these patients, and expansion to heritable and degenerative aortopathy, will be crucial in determining whether [18F]–sodium fluoride imaging could be used to stratify risk of adverse aortic outcomes more widely and subsequently to guide surgical candidacy.

## Notes

Supplement 1. **eTable 1.** Bicuspid Aortic Valve Only: Ascending Aortic [18F]–Sodium Fluoride (0.1 Increase) Linear Regression **eTable 2.** Bicuspid Aortic Valve Follow-up vs No Follow-up Demographics and Baseline Risk Factors for Aortopathy **eTable 3.** Linear Regression of Change in Ascending Aortic Diameter Over Time **eTable 4.** Associations Between Demographics, Cardiovascular Risk Factors, and Imaging Measurements When Stratified by Baseline Ascending Aortic Dilatation Status **eTable 5.** Ascending Aortic Circumferential Stiffness Index in Bicuspid Aortic Valve Linear Regression **eFigure.** CONSORT Diagram

Supplement 2.
Data Sharing Statement
