# Supplementary material for: Molecular Calcification Imaging and Ascending Aortic Disease in Patients With a Bicuspid Aortic Valve
Source: JAMA Netw Open. 2026 Feb 23;9(2):e2560385. doi: 10.1001/jamanetworkopen.2025.60385 (PMC12931472; doi:10.1001/jamanetworkopen.2025.60385)
Supplement: Supplement 1. — eTable 1. Bicuspid Aortic Valve Only: Ascending Aortic [18F]–Sodium Fluoride (0.1 Increase) Linear Regression eTable 2. Bicuspid Aortic Valve Follow-up vs No Follow-up Demographics and Baseline Risk Factors for Aortopathy eTable 3. Linear Regression of Change in Ascending Aortic Diameter Over Time eTable 4. Associations Between Demographics, Cardiovascular Risk Factors, and Imaging Measurements When Stratified by Baseline Ascending Aortic Dilatation Status eTable 5. Ascending Aortic Circumferential Stiffness Index in Bicuspid Aortic Valve Linear Regression eFigure. CONSORT Diagram [file jamanetwopen-e2560385-s001.pdf]

Supplementary Online Content

Nash J, Debono S, Loganath K, et al. Molecular calcification imaging and ascending aortic disease in bicuspid aortic valve. *JAMA Netw Open.* 2026;9(2):e2560385. doi:10.1001/jamanetworkopen.2025.60385

eTable 1. Bicuspid Aortic Valve Only: Ascending Aortic [18F]–Sodium Fluoride (0.1 Increase) Linear Regression

eTable 2. Bicuspid Aortic Valve Follow-up vs No Follow-up Demographics and Baseline Risk Factors for Aortopathy

eTable 3. Linear Regression of Change in Ascending Aortic Diameter Over Time

eTable 4. Associations Between Demographics, Cardiovascular Risk Factors, and Imaging Measurements When Stratified by Baseline Ascending Aortic Dilatation Status

eTable 5. Ascending Aortic Circumferential Stiffness Index in Bicuspid Aortic Valve Linear Regression

eFigure. CONSORT Diagram

This supplementary material has been provided by the authors to give readers additional information about their work.

© 2026 Nash J et al. *JAMA Network Open*.

eTable 1. Bicuspid Aortic Valve Only: Ascending Aortic [18F]–Sodium Fluoride (0.1 Increase) Linear Regression

Variable	B-estimate per 0.1 increase TBR_{mean} (95% confidence intervals)	p-value
**Age (per 10 years)**	1.09 (0.90-1.33)	0.36
**Male Sex**	1.15 (0.82-1.59)	0.42
**Hypertension**	0.98 (0.73-1.32)	0.91
**Coarctation**	0.98 (0.70-1.36)	0.90
**Antihypertensive Medication**	1.02 (0.76-1.36)	0.91
**Smoking status (compared to never smoker status**		
Ex smorker	0.98 (0.69-1.38)	0.89
Current smoker	1.03 (0.65-1.63)	0.91
**Weekly exercise**	0.88 (0.66-1.18)	0.40
**Diabetes Mellitus**	1.43 (0.68-2.98)	0.34
**Baseline Aortic Size index (mm/m^2)**	1.17 (0.74-1.85)	0.50
**Sex, age and body surface area adjusted ascending aortic aneurysm (95th centile, compared to no aneurysm)**	0.88 (0.64-1.20)	0.40
**Ascending aortic calcium score (Agaston units)**	1.00 (1.00-1.00)	0.56
**Aortic Stenosis (compared to no stenosis)**		
Mild	0.87 (0.62-1.21)	0.41
Moderate	1.05 (0.69-1.60)	0.82
**Aortic Regurgitation (compared to no regurgitation)**		
Mild	0.83 (0.57-1.20)	0.31
Moderate	0.92 (0.60-1.41)	0.71
Severe	1.17 (0.68-2.02)	0.57
**Bicuspid aortic valve morphology (compared to R+L morphology)**		
Right + Non-coronary leaflet fusion	0.90 (0.6-1.35)	0.60
Left + Non-coronary leaflet fusion	0.84 (0.24-3.02)	0.79
Two sinus type A	1.18 (0.75-1.86)	0.47
Two sinus type B	0.64 (0.26-1.60)	0.34

© 2026 Nash J et al. *JAMA Network Open*.

eTable 2. Bicuspid Aortic Valve Follow-up vs No Follow-up Demographics and Baseline Risk Factors for Aortopathy

	No follow up	Follow up	P value
**Number**	20	56	
**Age mean (SD), years**	52.97 (6.13)	52.40 (7.91)	0.77
**Male sex (%)**	15 (75.0)	42 (75.0)	1
**Bicuspid Aortic Valve subtype**			0.85
Right and left (%)	13 (65.0)	39 (69.6)	
Right and non-coronary (%)	4 (20.0)	8 (14.3)	
Left and non-coronary (%)	0 (0.0)	1 (1.8)	
Two sinus type A (%)	2 (10.0)	7 (12.5)	
Two sinus type B (%)	1 (5.0)	1 (1.8)	
**Stenosis Grade**			0.95
None (%)	12 (60.0)	32 (57.1)	
Mild (%)	5 (25.2)	16 (28.6)	
Moderate (%)	3 (15.0)	8 (14.3)	
**Regurgitation Grade**			0.49
None (%)	12 (60.0)	32 (57.1)	
Mild (%)	3 (15.0)	12 (21.4)	
Moderate (%)	2 (10.0)	9 (16.1)	
Severe (%)	3 (15.0)	3 (5.4)	
**Hypertension (%)**	8 (42.1)	24 (43.6)	1
**Diabetes (%)**	1 (5.3)	2 (3.6)	1
**Weekly Exercise (%)**	8 (42.1)	27 (49.1)	0.79
**Smoking**			0.82
Never	12 (63.2)	35 (63.6)	
Previous	4 (21.1)	14 (25.5)	
Current	3 (15.8)	6 (10.9)	
**Coarctation (%)**	5 (25.0)	14 (25.0)	1
**Hypertension Medication (%)**	8 (40.0)	28 (50.0)	0.61
**Ascending aortic [18F]sodium fluoride uptake median [IQR], tissue to background ratio**	1.10 [1.07, 1.14]	1.11 [1.06 to 1.14]	0.89
**Baseline Ascending Aortic Diameter median [IQR], mm**	37.85 [35.12 to 41.84]	38.47 [34.73 to 41.85]	0.79
**Baseline Ascending Aortic Size index median [IQR], mm/m^2**	17.82 [16.53 to 21.27]	19.11 [17.32 to 21.93]	0.19
**Ascending Aortic Calcium Score median [IQR], Agatston units**	0.00 [0.00 to 0.00]	0.00 [0.00 to 0.00]	0.84
**Ascending aortic dilatation (>95th centile after age, sex and body surface area adjustment)**	6 (30.0)	17 (30.4)	1

© 2026 Nash J et al. *JAMA Network Open*.

eTable 3. Linear Regression of Change in Ascending Aortic Diameter Over Time

	B-estimate per mm/year (95% confidence intervals	p-value	B-estimate per mm/year (95% confidence intervals	p-value
**Age (per 10 years)**	1.16 (0.94 – 1.43)	0.17		
**Hypertension**	1.12 (0.79 – 1.56)	0.51		
**Coarctation**	**0.68 (0.47 – 0.99)**	**0.045**	**0.68 (0.48 – 0.96)**	**0.031**
**Diabetes**	1.38 (0.55 – 3.45)	0.49		
**Hypertension Medication**	1.15 (0.82 – 1.61)	0.41		
**Male Sex**	0.95 (0.65 – 1.41)	0.81		
**Ascending Aortic Diameter**	1.00 (0.97 – 1.03)	0.88		
**Weekly Exercise**	1.24 (0.88 - 1.74)	0.21		
**Smoking**				
Previous smoking	0.99 (0.66 – 1.50)	0.98		
Current smoking	0.87 (0.49 – 1.54)	0.63		
**Ascending Aortic Calcium Score (Agaston units)**	0.95 (0.84 – 1.07)	0.39		
**Aortic Stenosis (Compared to none)**				
Mild	1.24 (0.84 – 1.83)	0.28		
Moderate	0.88 (0.53 – 1.46)	0.63		
**Aortic Regurgitation (compared to none)**				
Mild	1.08 (0.70 – 1.67)	0.72		
Moderate	1.37 (0.85 – 2.29)	0.20		
Severe	1.23 (0.48 – 3.13)	0.66		
**Bicsupid Aortic Valve Subtype (compared to R+L subtype)**				
Right and Non-coronary cusp Fusion	1.12 (0.68 – 1.85)	0.64		
Right and Left coronary cusp fusion	0.56 (0.15 – 2.08)	0.38		
Two sinus type A	0.96 (0.56 – 1.62)	0.86		
Two sinus type B	1.34 (0.36 – 4.95)	0.66		
**Ascending Aortic Sodium Fluoride TBRmean (per 0.1 increase)**	**0.70 (0.55 – 0.90)**	**0.005**	**0.70 (0.55 – 0.88)**	**0.004**

© 2026 Nash J et al. *JAMA Network Open*.

eTable 4. Associations Between Demographics, Cardiovascular Risk Factors, and Imaging Measurements When Stratified by Baseline Ascending Aortic Dilatation Status

	**No Aortic Dilatation (n=53)**	**Aortic Dilatation (n=23)**	**p-value**
**Age mean (SD), years**	53.0 (7.4)	51.4 (7.6)	0.39
**Male Sex (%)**	44 (83.0)	13 (56.5)	0.031
**Family History of Aortic Dissection (%)**	3 (5.9)	0 (0.0)	0.58
**Bicuspid Aortic Valve Subtype**			0.54
Right/Left Fusion (%)	39 (73.6)	13 (56.5)	
Right/Non-Coronary Cusp Fusion (%)	7 (13.2)	5 (21.7)	
Left/Non-Coronary Cusp Fusion (%)	1 (1.9)	0 (0.0)	
Two Sinus Type A (%)	5 (9.4)	4 (17.4)	
Two Sinus Type B (%)	1 (1.9)	1 (4.3)	
**Aortic Stenosis Grade**			0.044
None (%)	28 (52.8)	16 (69.6)	
Mild (%)	19 (35.8)	2 (8.7)	
Moderate (%)	6 (11.3)	5 (21.7)	
**Aortic Regurgitation Grade**			0.70
None (%)	32 (60.4)	12 (57.1)	
Mild (%)	10 (18.9)	5 (23.8)	
Moderate (%)	8 (15.1)	3 (13.0)	
Severe (%)	3 (5.7)	3 (13.0)	
**Hypertension (%)**	24 (47.1)	8 (34.8)	0.46
**Diabetes (%)**	3 (5.9)	0 (0.0)	0.58
**Weekly Exercise (%)**	25 (49.0)	10 (43.5)	0.85
**Smoking Status**			0.65
Never (%)	31 (60.8)	16 (69.6)	
Previous smoker (%)	14 (27.5)	4 (17.4)	
Current (%)	6 (11.8)	3 (13.0)	
**Coarctation (%)**	14 (26.4)	5 (21.7)	0.89
**Hypertension medication (%)**	28 (52.8)	8 (34.8)	0.23
**Ascending aortic [18F]sodium fluoride uptake median [IQR], tissue to background ratio**	1.12 [1.06, 1.14]	1.09 [1.06, 1.12]	0.33
**Baseline Ascending Aortic Diameter median [IQR], mm**	36.09 [33.75, 38.91]	44.06 [41.48, 46.64]	<0.001
**Baseline Ascending Aortic Size index median [IQR], mm/m^2**	17.57 [16.39, 19.11]	22.69 [21.94, 24.00]	<0.001
**Ascending Aortic Calcium Score median [IQR], Agatston units**	0.00 [0.00, 0.00]	0.00 [0.00, 0.00]	0.092
**Annualized diameter change median [IQR], mm/year**	0.66 [0.13, 0.94]	0.61 [0.25, 0.71]	0.87

© 2026 Nash J et al. *JAMA Network Open*.

Mean (+/- standard deviation), Median [Interquartile Range], * T-Test for normally distributed data, Wilcoxon for non-parametric data, Chi-squared for categorical data

© 2026 Nash J et al. *JAMA Network Open*.

eTable 5. Ascending Aortic Circumferential Stiffness Index in Bicuspid Aortic Valve Linear Regression

Variable	B-estimate per 10 SI units increase (95% confidence intervals)	P value	B-estimate per 10 SI units increase (95% confidence intervals	P value
**Age (per 10 years)**	**1.44 (1.18 – 1.75)**	**<0.001**	**1.36 (1.14 – 1.62)**	**<0.001**
**Hypertension**	1.10 (0.80 – 1.51)	0.54		
**Coarctation**	1.20 (0.83—1.73)	0.32		
**Diabetes**	**3.26 (1.56-6.81)**	**0.002**	**2.31 (1.21 – 4.43)**	**0.012**
**Hypertension Medication**	1.06 (0.77-1.45)	0.71		
**Male Sex**	0.89 (0.62-1.28)	0.52		
**Ascending Aortic Diameter**	**1.03 (1.00-1.06)**	**0.035**	**1.03 (1.01 – 1.06)**	**0.009**
**Weekly Exercise**	0.85 (0.62-1.17)	0.32		
**Smoking**				
Previous smoking	1.07 (0.74-1.53)	0.73		
Current smoking	0.68 (0.41-1.08)	0.098		
**Ascending Aortic Calcium Score (Agaston units)**	1.00 (1.00 – 1.00)	0.072		
**Aortic Stenosis (Compared to none)**				
Mild	0.80 (0.55-1.15)	0.23		
Moderate	0.90 (0.56-1.46)	0.67		
**Aortic Regurgitation (compared to none)**				
Mild	0.70 (0.46-1.06)	0.089		
Moderate	0.63 (0.39-1.00)	0.052		
Severe	0.95 (0.50-1.78)	0.86		
**Bicsupid Aortic Valve Subtype (compared to R+L subtype)**				
Right and Non-coronary cusp Fusion	0.75 (0.49-1.15)	0.19		
Right and Left coronary cusp fusion	1.47 (0.38-5.61)	0.57		
Two sinus type A	1.48 (0.92-2.40)	0.11		
Two sinus type B	0.59 (0.23-1.54)	0.28		
**Ascending Aortic Sodium Fluoride TBRmean (per 0.1 increase)**	**1.51 (1.19 – 1.90)**	**<0.001**	**1.33 (1.08 – 1.62)**	**0.007**

© 2026 Nash J et al. *JAMA Network Open*.

eFigure. CONSORT Diagram

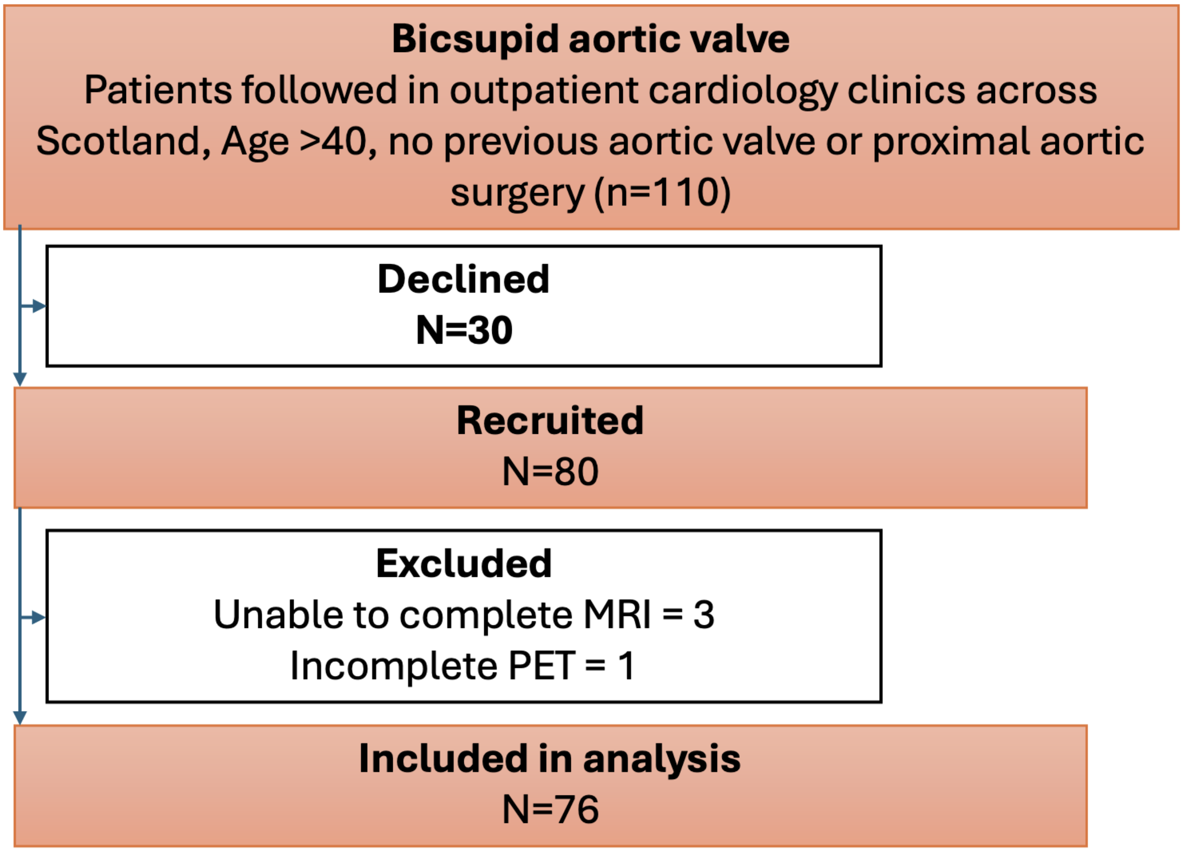

© 2026 Nash J et al. *JAMA Network Open*.
