# Supplementary material for: Molecular Calcification Imaging and Ascending Aortic Disease in Patients With a Bicuspid Aortic Valve
Source: JAMA Netw Open. 2026 Feb 23;9(2):e2560385. doi: 10.1001/jamanetworkopen.2025.60385 (PMC12931472; doi:10.1001/jamanetworkopen.2025.60385)
Supplement: Supplement 2. — Data Sharing Statement [file jamanetwopen-e2560385-s002.pdf]

Data Sharing Statement

Nash. Molecular Calcification Imaging and Ascending Aortic Disease in Patients With a Bicuspid Aortic Valve. *JAMA Netw Open*. Published February 23, 2026.
doi:10.1001/jamanetworkopen.2025.60385

Data
Data available: Yes
Data types: Deidentified participant data
How to access data: De-identified patient data will be made available following reasonable request to the senior author alexander.fletcher.2@glasgow.ac.uk.
When available: With publication

Supporting Documents
Document types: None

Additional Information
Who can access the data: Researchers whose proposed use of the data has been approved
Types of analyses: The code for each of the analyses will be made available upon reasonable request
Mechanisms of data availability: With a signed data access agreement
